# Effect of a multi-ingredient based food supplement on sexual function in women with low sexual desire

**DOI:** 10.1186/s12905-019-0755-9

**Published:** 2019-04-30

**Authors:** S. Palacios, E. Soler, M. Ramírez, M. Lilue, D. Khorsandi, F. Losa

**Affiliations:** 1 Gynaecology and Obstetrics Department, Palacios’ Institute of Women’s Health, C/Antonio Acuña 9, E-28009 Madrid, Spain; 2 Procare Health Iberia, Medical department, Barcelona, Spain; 3 University of Barcelona, Barcelona, Spain; 4 Clínica de la Sagrada Familia, Barcelona, Spain

**Keywords:** Sexual function, Low sexual desire, *Trigonella foenum graecum*, Testosterone

## Abstract

**Background:**

Studies have demonstrated that women with low desire and low excitement have negative feelings regarding their physical and emotional satisfaction, as well as their happiness. In this study, we evaluate the efficacy of Libicare® - a multi-ingredient food supplement - to improve sexual function in postmenopausal women.

**Methods:**

This was an exploratory, prospective, non-controlled, observational study. Postmenopausal women aged 45–65 with a risk of sexual dysfunction (Female Sexual Function Index (FSFI) < 25.83) were included during routine clinical visits and treated with 2 tablets of Libicare® daily for 2 months. Libicare® is an oral food supplement containing *Trigonella foenum graecum*, *Turnera diffusa*, *Tribulus terrestris*, and *Ginkgo biloba* dry extracts. Primary endpoint: change vs. baseline in FSFI score. Secondary endpoints: 1) changes in testosterone and serum steroid levels of free testosterone and sex hormone-binding globulin (SHBG) levels and 2) tolerability.

**Results:**

A total of 29 patients (mean age: 54.69 years) were included. FSFI mean (SD) score showed a significant increase: 20.15 (4.48) vs 25.03 (6.94), baseline vs final; *p* = 0.0011, paired t-test. Most patients (86.2%) increased their FSFI score. All FSFI domains, except dyspareunia, showed significant increases. The highest increase was observed in the desire domain (*p* = 0.0004). Testosterone and SHBG levels were assessed in 21 patients. A significant increase in testosterone level was observed: 0.41 (0.26) vs. 0.50 (0.34) pg/mL, baseline vs. final; *p* = 0.038, Wilcoxon test. 52.4% of patients increased their testosterone levels. Finally, a significant decrease was observed in SHBG level: 85 (32.9) vs. 73 (26.8) nmol/L, baseline vs. final; *p* = 0.0001; paired t-test. 95.2% of patients decreased their SHBG levels.

**Conclusion:**

In this pilot study, a significant improvement in sexual function and related hormone levels was observed with Libicare®. Further studies must be conducted to confirm these exciting results.

**Trial registration:**

Current Controlled Trial ISRCTN12928573. Date of registration: 28/March/2019. Retrospectively registered.

## Background

Low sexual desire affects more than 20% of women [1]. A survey among American and European women (Women’s International Study of Health and Sexuality, WISHeS) evaluated the prevalence of hypoactive sexual desire [2, 3]. Among European women, aged 20–49, in their reproductive age, the prevalence of reduced desire causing discomfort and concern was 7%. In women aged 50–70, the prevalence was 9% in women with natural menopause, and 12% in women subjected to surgical menopause [2].

After interviewing 750 women, the most common sexual issues described in the Spanish population were lack of sexual interest (36.0%), impossibility of having an orgasm (27.8%), and unsatisfactory sexual relationships (25.1%) [4].

Women with low sexual desire may have problems when starting or having stable sexual relationships, and they may feel unsatisfied and experience marital disorders. Studies have demonstrated that women with low desire, low excitement, or sexual pain are clearly associated with negative feelings regarding their physical and emotional satisfaction, as well as their happiness. In addition, women suffering from those problems tend to experience much more negative emotions and psychological states than women with normal sexual activity [5, 6].

The 5th edition of the Diagnostic Statistical Manual of Mental Disorders, (DSM-V, 2013) [7] features some changes as an attempt to correct, extend, and clarify the different diagnoses related to sexual dysfunction and their criteria. For instance, they have brought female desire and excitement disorders together under a single diagnosis called ‘Female Sexual Interest/Arousal Disorder’.

Biological, psychological, and interpersonal factors play a part in sexual dysfunction. The hormones impacting female sexuality (estrogens and androgens) play a crucial role in sexual function [8]. Various studies have demonstrated that reduced testosterone levels are directly related to decreased sexual activity in postmenopausal women [9, 10]. However, the fact that women produce higher amounts of androgens than estrogens demonstrates how important their role is. Having a deeper knowledge of intracrinology may potentially facilitate a better understanding of the correlation between androgen levels and sexual desire [11].

There is clear clinical evidence that strongly suggests that androgens play a vital role in sexual dysfunction, taking into account the findings published on *Trigonella* regarding increased testosterone blood levels and improved sexual function and desire [12–14]; as well as the potentially beneficial effects on sexual function published on *Turnera diffusa* as a regulator of the natural balance between androgens and estrogens [15–17]; in addition to *Tribulus terrestris* [18] and *Ginkgo biloba* [15–17] to facilitate blood flow and to provide a relaxing effect on smooth muscle. Overall, studying the effect of an extract with all these components on women with low sexual desire was deemed necessary. We presented this data at the 21st World Meeting on Sexual Medicine as a poster [https://www.sciencedirect.com/science/article/pii/S1743609518304399?via%3Dihub].

Therefore, the objective of this pilot study is to evaluate the efficacy of a multi-ingredient food supplement, marketed under the trade name Libicare®, to improve sexual function in postmenopausal women.

## Methods

This is an observational, prospective, non-controlled pilot study scheduled for 30 postmenopausal participants aged 45–65 with a Female Sexual Function Index (FSFI) < 25.83, this number being considered as the threshold for low sexual dysfunction risk [19]. All women were included at routine clinical visits and

were treated with 2 tablets of Libicare®, one every 12 h, daily for 2 months (9 weeks). Libicare® is an oral food supplement containing dry extracts of *Trigonella foenum graecum*, *Turnera diffusa*, *Tribulus terrestris*, and *Ginkgo biloba* (see Table 1). Libicare® is manufactured by Procare Health (Barcelona, Spain). The primary endpoint was to evaluate the efficacy on sexual function. Risk of sexual dysfunction was evaluated using the FSFI. The FSFI is a 19-item questionnaire divided into six domains: desire, arousal, lubrication, orgasm, satisfaction, and pain [20–23]. Scoring system: the individual score was achieved and then added to other scores from the same domain, multiplied by the relevant factor. The total scale was achieved by adding the scores obtained in the 6 domains. This tool provides optimal psychometric properties for each of the 6 domains, is easy to use, and has been demonstrated to be able to discriminate between clinical populations (women with risk of sexual dysfunction) and non-clinical populations (without sexual disorders).

**Table 1:** Libicare® nutritional information

Nutritional Information	For 2 tablets	Nutrient Reference Value (NRV)
Dried extract of Trigonella ˗ Saponines	600 mg 300 mg	- -
Dried extract of Damiana	150 mg	–
Dried extract of Ginkgo ˗ Ginkgo flavonoids	50 mg 12.50 mg	- -
Dried extract of Tribulus ˗ Saponins	40 mg 36 mg	- -
Vitamin B3	32 mg	200
Vitamin B2	2.8 mg	200
Vitamin B1	2.2 mg	200
Vitamin B6	2 mg	143
Selenio	50 μg	90.91

A selection period during which other inclusion criteria apart from the FSFI index were also evaluated was followed by a 9-week treatment period.

### Inclusion criteria

- Healthy, postmenopausal women (no natural menses for at least 1 year) aged ≥45 and ≤ 65. Hysterectomized patients should have an FSH level above 40 IU.
- Women with a stable partner, living together for at least 15 days a month and being sexually available.
- Risk of sexual dysfunction established at FSFI score < 25.83.
- Integrity of the vaginal mucosa (without lesions or bleeding).
- Women willing to and capable of understanding and signing an informed consent after receiving an explanation on the nature of the whole study.
- Consenting to participate in the study and signing the Informed Consent form.
- No desire for pregnancy in the next 3 months.

### Exclusion criteria

- Non-compliance with the requirements above.
- Pregnant women or with suspected pregnancy.
- Within 3 months following delivery or abortion.
- Breastfeeding women.
- Women with severe pain in sexual relationships (DMS-V).
- Non-diagnosed abnormal genital bleeding or presence of vaginal lesions.
- Women with symptoms of vaginal infection or signs of any other genital infection.
- Women allergic or hypersensitive to the components of the study treatment.
- Severe psychiatric disorder.
- Use of any hormonal treatment with estrogens, progestogens, or estrogens and progestogens within 3 previous months prior to selection.
- Use of any other drug or experimental device within 30 days prior to selection.
- Any condition preventing the patient from participating in the study, at the researcher’s discretion.

All participants received a written consent form and signed it before any procedure related to the study was carried out. Participants underwent a medical history review, medical examination, and comprehensive gynecological check-up. A gynecological examination was performed to assess the appearance of the mucosa and the tolerance to medication on day 1 and on week 9.

### Laboratory tests

Serum steroid levels of free testosterone and sex hormone-binding globulin (SHBG) were measured in the LABCO Laboratory. SHBG was measured with chemiluminescent immunoassay Immulite 2000 XPi (Siemens Healthcare Diagnostics, Eschborn, Germany) with an inter-assay coefficient of variation of 3.5 and 8.3% at the low level, and 4.8 and 5.4% at the high level, respectively. The estimation of serum free testosterone hormone levels was carried out using the ELISA technique. The DiaMetra Italy kit (DKO-015) was used to determine free testosterone hormone concentration in human serum according to the manufacturer’s instructions.

FSFI was performed in the whole sample (29 individuals), and determinations of free testosterone and SHBG were achieved in 21 individuals [20]. Hormonal determination was only performed in patients giving their consent to that. The characteristics of the analytical parameters (FSFI free testosterone and SHBG) in the two visits (pre/post), the absolute change between visits (difference post-pre), and the relative change between visits ([post-pre]/pre × 100%) were described.

### Statistical methodology

A descriptive analysis of the variables included in the study was carried out. Central tendency and dispersion measures were presented, that is, the mean, standard deviation (SD), median, 25th and 75th percentiles (P25 and P75, respectively), and minimum and maximum (min. and max., respectively) of quantitative variables.

Absolute (n) and relative (%) frequency distributions of qualitative variables were presented. Unavailable data were not allocated. They were just described as lost data.

To analyze the evolution/change throughout the study, parametric tests for continuous variables (Student’s T test for paired samples) and/or non-parametric tests (Wilcoxon) were used.

The hypothesis testing was two-tailed in all cases, with a significance level of *p* < 0.05. Analyses were carried out using the SAS statistical software, version 9.4.

## Results

The sample to be analyzed was made up of 29 patients who completed the study. One patient withdrew from the study as she had to travel. Of the 29 patients, 21 consented to blood tests for hormonal determination prior to and upon completion of the study.

Demographic data from patients are shown in Table 2. Mean age was 54.69 (45–65). All women were Caucasian and from varied educational and economic backgrounds. In this study group, all women were considered as healthy based upon clinical history, examination, and recent blood and biochemistry tests less than 3 months old.

**Table 2:** Demographic characteristics (*n* = 29)

Age (years, mean SD)	54.69 (6.29)
Weight (Kg, mean SD)	63.33 (8.81)
Height (cm, mean SD)	159.40 (7.06)
Academic level (n, %)	
Primary	11 (39.2%)
Secondary	8 (28.5%)
Graduated	9 (32.3%)
N/A	1
Gynecological characteristics	
Mean menarche age (SD)	12.62 (1.5)
Mean menopause age (SD)	49.42 (6.92)
Hysterectomy n%	4 (13.8%)
Mean pregnancies	1.23 (1.06)
Mean deliveries (SD)	1.03 (1.09)

**Fig. 1:**
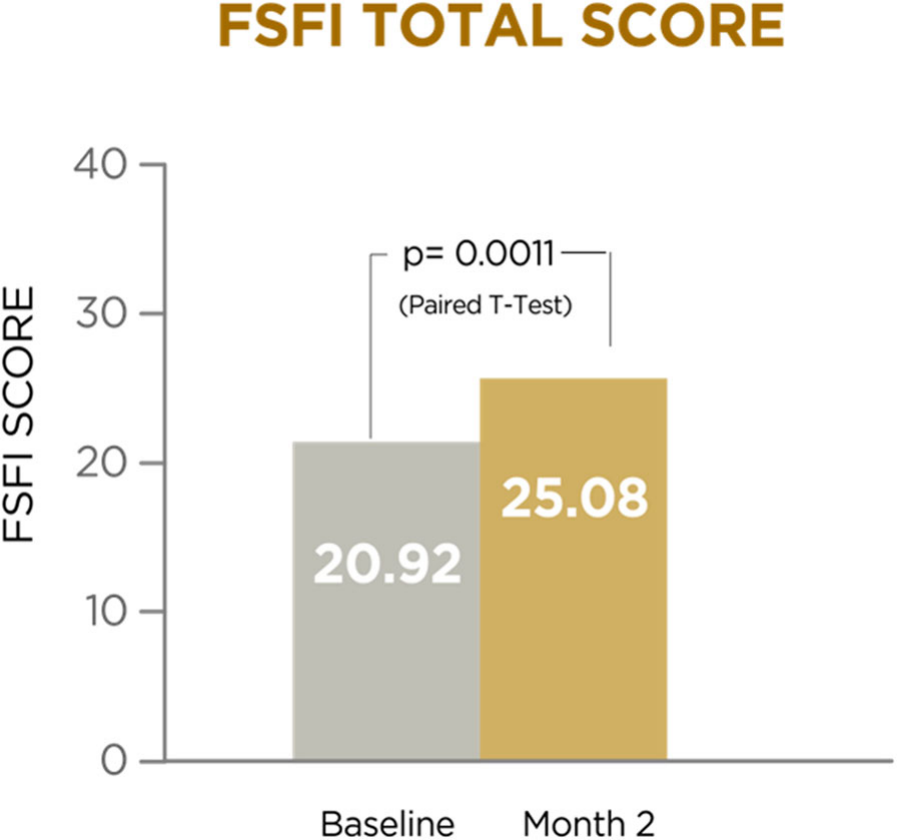
FSFI total score at baseline and after 2 months of Libicare

FSFI results are shown in Table 3 and Fig. 1, which features the mean of baseline values and values after two months, with their standard deviations, for each domain, as well as the global determination. As demonstrated by the results after treatment, values were significant for all domains except for dyspareunia. The domain presenting the highest increase was desire, with *p* = 0.0004. The global domain increased 4.15 (6.14) points on average, with a statistical significance of *p* = 0.0011.

**Table 3:** Mean of baseline values and values after two months of Libicare® treatment. Absolute change and statistical significance of the different FSFI domains

	Mean at baseline	Mean at 2 months	Absolute change (mean)	SS
Desire	2.40 (0.96)	3.33 (1.29)	0.93 (1.25)	*p* = 0.0004
Excitement	3.04 (0.96)	3.63 (1.44)	0.59 (1.25)	*p* = 0.0166
Lubrication	3.70 (0.98)	4.50 (1.34)	0.80 (1.34)	*p* = 0.0034
Orgasm	3.57 (1.14)	4.23 (1.43)	0.66 (1.30)	*p* = 0.0106
Sexual satisfaction	4.01 (0.99)	4.62 (1.22)	0.61 (1.45)	*p* = 0.0321
Dyspareunia	4.19 (1.06)	4.76 (1.40)	0.57 (1.55)	*p* = 0.0602
Global	20.42 (4.23)	25.08 (6.62)	4.15 (6.14)	*p* = 0.0011

There were 18 patients, accounting for 62% of subjects, who initially had sexual dysfunction risk scores and eventually reached normality, with their FSFI increasing beyond 25.83. A total of 86.2% of patients experienced an increase in their FSFI following treatment (Fig. 1).

**Fig. 2:**
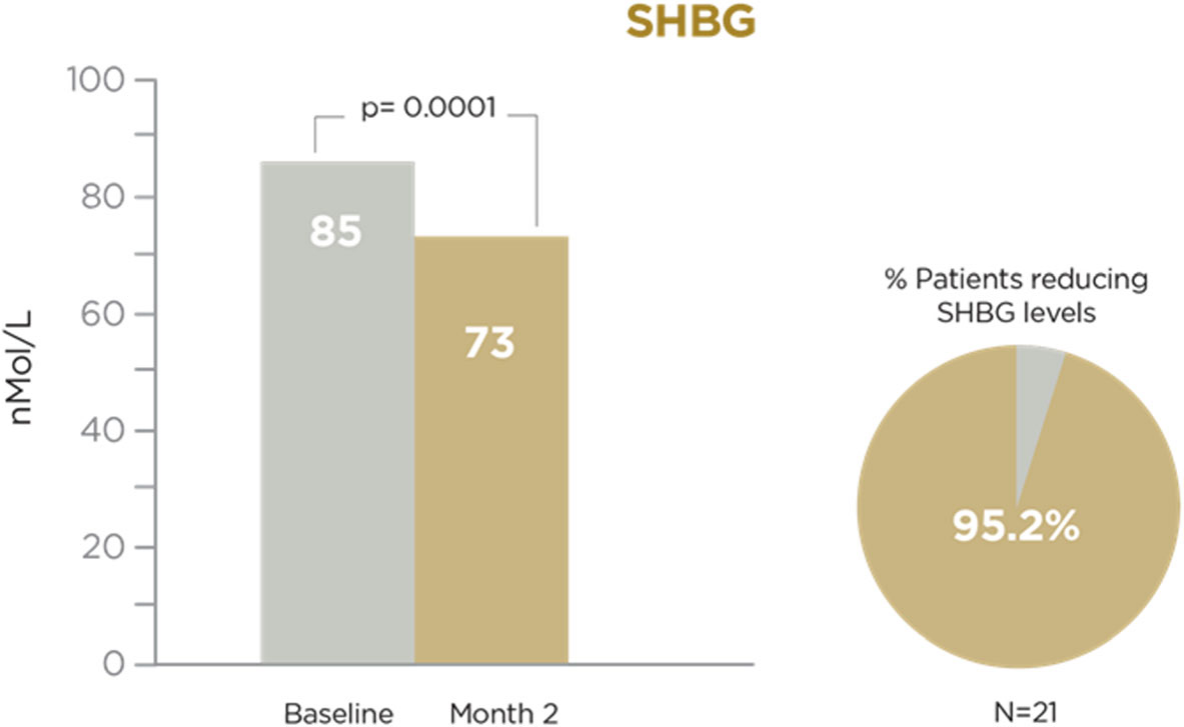
Free testosterone at baseline and after 2 months of Libicare

As far as free testosterone data are concerned, Fig. 2 shows the numbers at visit 1 (pre) and visit 2 (post), as well as the change between both visits. With a sample of 21 patients with available data, statistically significant differences were noted in testosterone values between visit 1 (pre) and visit 2 (post). Testosterone mean change between visits (post-pre) was an increase of 0.09 (SD 0.17) units (*p* = 0.0386); the mean percent change as compared to baseline was 79.3% (*p* = 0.0214).

**Fig. 3:**
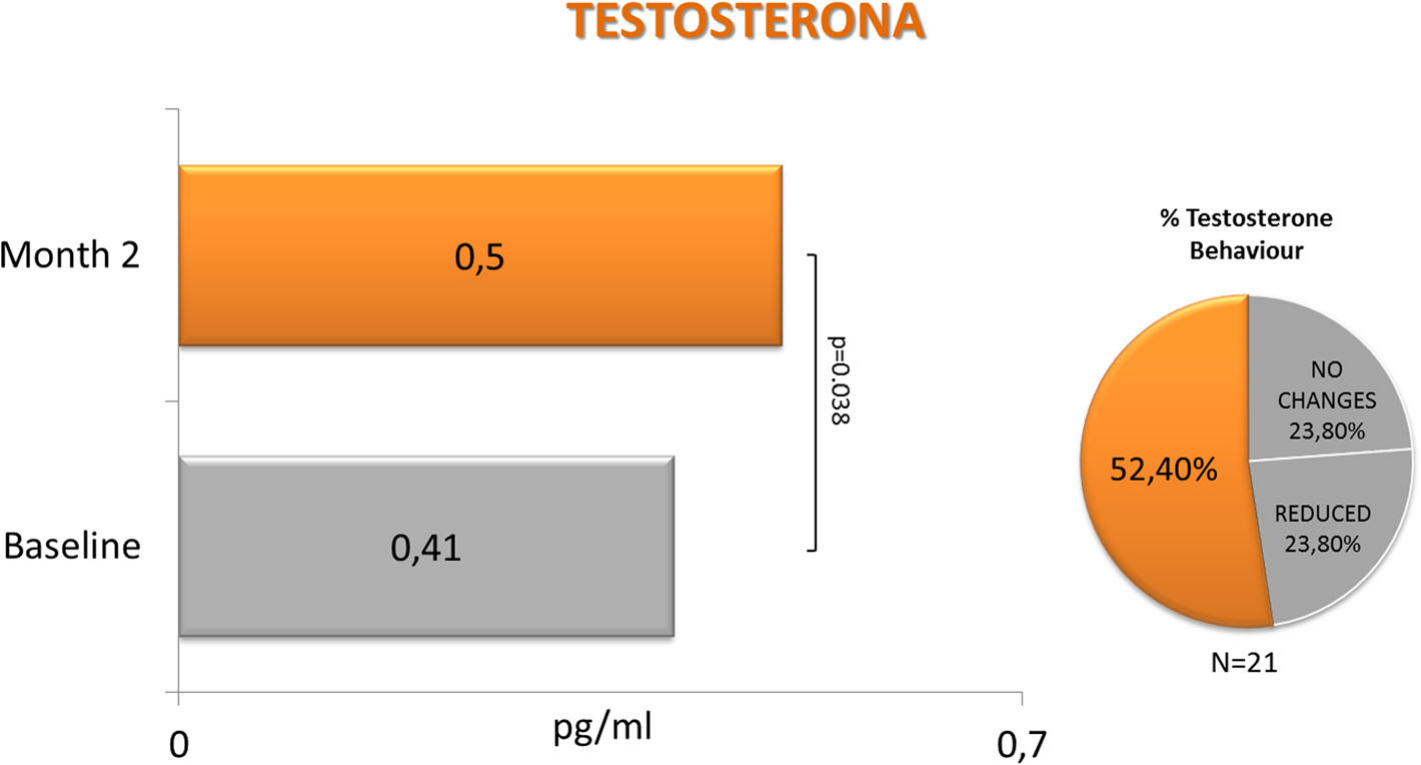
SHBG at baseline and after 2 months of Libicare

Finally, SHBG data at visit 1 (pre) and visit 2 (post), as well as changes between both visits, are shown in Fig. 3. With a sample of 21 patients with available data, statistically significant differences were noted in SHBG values between visit 1 (pre) and visit 2 (post). The mean SHBG change between visits (post-pre) represented a decrease of − 12.05 (SD 11.48) units (*p* = 0.0001); the mean percent change as compared to baseline was − 13.17% (*p* < 0.0001). 95.2% of patients assessed experienced a decrease in SHBG values (only one patient had increased SHBG values).

## Discussion

The hormones that influence female sexuality are estrogens and androgens. Several studies have shown that a reduction in testosterone level is directly related to a decrease in sexual activity in postmenopausal women [9, 10]. However, it is also true that other studies have shown that the level of testosterone is not related to sexual performance [24, 25]. On the other hand, there is increasing data pointing to a possible role of androgens on the neurotransmitter dopamine and serotonin [26, 27]. Dopamine plays an essential role in the modulation of sexual desire; it seems to increase sexual desire, the subjective sensation of excitement and the desire to continue sexual activity once the stimulation has begun [27, 28].

Although the fact of testosterone’s effectiveness as a treatment in female sexual interest disorders has already been demonstrated, there are some doubts regarding its long-term safety [29–32]. This is the reason that the US Food and Drug Administration avoided authorizing testosterone patches to be marketed [33] and paving the way for new strategies to fight this disorder.

One of them is flibanserin, a 5HT1_a_ agonist and a 5HT2_a_ antagonist, which has been demonstrated to increase sexual desire, reduce sexual dysfunction-associated discomfort, and improve sexual activity in premenopausal women with low sexual desire, with a good tolerability [34, 35], which translated into flibanserin being approved in the US [36]. However, this central nervous system drug also has some side effects, which has led to some controversy [37], naturally paving the way for new products to treat and improve low female sexual interest.

Therefore, investigating new therapeutic options based on safe natural products which have been widely used for decades and have been demonstrated as effective for sexual desire and arousal seems more than reasonable.

The results from this study, using a multi ingredient-based product including *Trigonella foenum graecum, Turnera diffusa*, *Tribulus terrestris*, and *Ginkgo biloba*, made us think it may have a potential double effect, improving sexual desire through an increase in free testosterone, but also improving arousal by means of a vaginal vasodilating effect. All these effects with the separate components are widely described in numerous studies [12–18], https://www.sciencedirect.com/science/article/pii/S1743609518304399?via%3Dihub.

In our study, the whole array of natural components Libicare® consists of has demonstrated a statistically significant improvement in desire, arousal, lubrication, orgasm, and sexual satisfaction domains. Indeed, the global analysis of all FSFI domains demonstrated a significant improvement of more than 4 points after 2 months of treatment,

Dyspareunia was the only domain showing improvement without statistical significance. This was to be expected as one of the exclusion criteria in this study was severe pain in sexual relationships. Owing to its characteristics, this product can be used to address female desire and arousal disorders, that is, the Female Sexual Interest/Arousal Disorder described in the DSM-V [7]. In addition, dyspareunia domain data are close to significance after 2 months of treatment. If used longer, the improvement could possibly achieve statistical significance.

Most patients improved their FSFI score following 2 months of Libicare® treatment with 62% of them achieving normality rates. Only 13.8% of women had no improvement whatsoever or a decrease in FSFI score. Even though this is a small percentage, this possibly demonstrates the relevance of the multifactorial causes of this disorder and how important treatment selection and individualization is.

As we already said, this improvement with the use of *Trigonella foenum graecum*, which proves especially significant regarding sexual desire, had already been found by other authors. Indeed, Amanda Rao et al. [14] demonstrated that a specialized extract of *Trigonella foenum graecum* seed has a positive impact on the improvement of sexual function, specifically in both sexual desire and arousal, in healthy menstruating women with low sexual function. In our case, patients were postmenopausal and older (45–65), with equally positive results. Furthermore, Elham Aktari et al. [38], used *Tribulus terrestris* in women with hypoactive sexual desire, and after 4 weeks, they found statistically positive effects on the FSFI score.

The significant increase observed in free testosterone consolidates the results and explains the rise of sexual desire. It is worth noting that this increase is modest and within normal levels, and it is also correlated with the significant decrease found in SHBG levels. Both free testosterone and SHBG hormonal changes have already been demonstrated with *Trigonella foenum-graecum* extracts by other authors [14].

This pilot study has two limitations: a small number of women included and no control or placebo group. Therefore, these clinical results should be reinforced with other randomized, placebo-controlled studies. On the other hand, the small size of the sample does not allow knowing the importance of these results.

The administration of this product is associated with a significant increase not only in desire and arousal, but also in vaginal lubrication and orgasm. As previously explained, this occurs as part of a domino effect which makes other sexual domains improve when one of them does, and also as a double effect of this product on increasing free testosterone and local vasodilatation. The effect and activity of *Turnera diffusa* and *Ginkgo biloba*, the first with an anti-aromatase activity [39], and the second with a vasodilation activity [40], account for the improvement in these two domains (lubrication and orgasm).

There are very few studies analyzing the effect of *Trigonella foenum-graecum* on female sexual function. The results from our work support others previously found [14]. Additionally, the possibilities of the various components of this product pave the way to understand the effects on the different domains of sexuality and the double effect, both on desire and arousal.

## Conclusion

In this pilot study, the multi-ingredient food supplement, Libicare®, has shown an improvement in desire, arousal, lubrication, orgasm, and sexual satisfaction domains, with a clear increase in free testosterone numbers and a decrease in SHBG levels in postmenopausal women. It is necessary to perform a randomized study with a control group to confirm these results.

## Abbreviations

DKO-015: DiaMetra Italy kit
DSM: Diagnostic Statistical Manual of Mental Disorders
FSFI: Female Sexual Function Index
SD: Standard deviation
SHBG: Sex hormone blinding globulin
